# Secretion of flagellin by the LEE-encoded type III secretion system of enteropathogenic *Escherichia coli*

**DOI:** 10.1186/1471-2180-9-30

**Published:** 2009-02-06

**Authors:** Luminita Badea, Scott A Beatson, Maria Kaparakis, Richard L Ferrero, Elizabeth L Hartland

**Affiliations:** 1 Department of Microbiology, Monash University, Melbourne, Victoria, Australia; 2 Department of Microbiology and Immunology, University of Melbourne, Melbourne, Victoria, Australia; 3 School of Molecular and Microbial Sciences, University of Queensland, Brisbane, Queensland, Australia

## Abstract

**Background:**

Enteropathogenic *Escherichia coli *(EPEC) is an attaching and effacing (A/E) pathogen that possesses a type III secretion system (T3SS) encoded within the locus of enterocyte effacement (LEE). The LEE is essential for A/E lesion formation and directs the secretion and translocation of multiple LEE-encoded and non-LEE encoded effector proteins into the cytosol of infected cells. In this study we used proteomics to compare proteins exported to the culture supernatant by wild type EPEC E2348/69, a Δ*espADB *mutant and a Δ*escF *T3SS mutant.

**Results:**

We observed that flagellin was consistently and strongly present in the secretome of wild type EPEC and the Δ*espADB *mutant but present only weakly in the secretome of the Δ*escF *mutant. Given the ancestral relationship between the flagella export apparatus and virulence associated T3SSs, we investigated whether FliC could utilise the LEE-encoded T3SS for export. In the absence of a functional flagella export apparatus, we showed that FliC could be secreted by the LEE-encoded T3SS and stimulate (Toll-like receptor 5) TLR5 signalling but could not confer motility.

**Conclusion:**

Since the secretion of FliC during A/E lesion formation would presumably be disadvantageous for the pathogen, we propose that virulence associated T3SSs and flagella T3SSs have evolved through a system of chaperones and complex regulatory pathways to be functional at different times to ensure that FliC secretion does not occur during T3SS effector translocation.

## Background

EPEC is an important cause of infant diarrhea in the developing world and is one of several gastrointestinal pathogens of humans and animals capable of causing distinctive lesions in the gut, termed attaching and effacing (A/E) lesions [1-3]. A/E lesions are manifested by damage to the integrity of the enterocyte cytoskeleton, which involves intimate attachment of the bacteria to the cell surface coincident with the formation of actin rich pedestal-like structures underneath tightly adherent bacteria [4]. A/E lesion formation is mediated by proteins encoded within a large pathogenicity island called the locus of enterocyte effacement (LEE) [5], which is essential for A/E lesion formation and highly conserved among A/E pathogens [6,7]. The LEE encodes regulators, a type III secretion system (T3SS), T3SS chaperones as well as secreted translocator and effector proteins [5,8,9]. The T3SS itself is a multiprotein needle-like complex evolutionarily related to the flagella apparatus that comprises more than 20 proteins spanning both the inner and outer membranes of the bacterial envelope. The T3SS secretes and translocates virulence effector proteins from the bacterial cytosol directly into the host cell cytoplasm, where the effector proteins facilitate disease development [10]. Structurally the needle complex closely resembles a flagella basal body [11,12], supporting an evolutionary relationship between the flagella export apparatus and T3SSs. However, despite the architectural similarity between the flagella biosynthesis machinery and T3SSs, the structural components of the needle complex share limited sequence similarity with components of the flagella basal body [12,13].

A unique feature of the EPEC LEE-encoded T3SS is the presence of a filamentous structure formed by monomers of EspA that connect the EscF T3SS needle to the pore forming translocation proteins, EspB and EspD [8,14]. EspA demonstrates discrete sequence similarity to flagellin in the carboxyl-terminal region of the protein which is predicted with high probability to adopt a coiled-coil conformation [15,16]. Similar to the assembly of flagella from the polymerization of monomeric flagellin [17], polymerization of EspA to form filaments depends on coiled-coil interactions between EspA subunits [15]. In addition, it has been shown that EspA subunits are added to the tip of the growing filament in a similar manner to a growing flagellum [18]. Although EspA filament diameter (120 Å) is smaller than that of flagella (230 Å), its assembly has a lumen diameter and helical symmetry parameters very similar to those of the flagellar filamentous structure [13,19,20]. Despite these structural similarities, to date no functional overlap has been observed between the two protein secretion systems in EPEC. In this study, we observed that FliC was consistently present in the secretome of wild type EPEC E2348/69 or an Δ*espADB *mutant of E2348/69 but only weakly present in the secretome of a Δ*escF *(T3SS) mutant of EPEC E2348/69. We determined that FliC could be secreted by the LEE-encoded T3SS of EPEC E2348/69 and that FliC exported in this manner was able to stimulate an inflammatory response via the pathogen-recognition molecule for bacterial flagellin, Toll-like receptor 5 (TLR5).

## Results

### Analysis of the EPEC E2348/69 secretome

**Figure 1:**
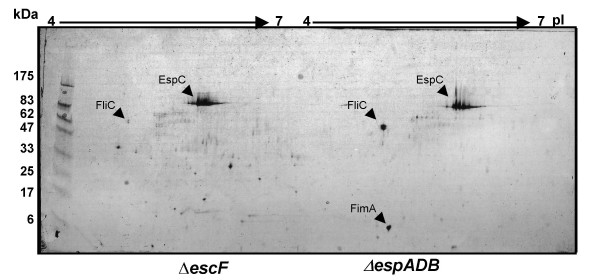
**Comparative 2-DGE analysis of the secretomes of EPEC E2348/69 derivatives, ICC171 Δ*escF *and Δ*espADB***. Protein secretion was induced by growth of the culture to OD_600 _1.0 in hDMEM. Protein size markers are shown in kDa and pI values (IPG strips of 4–7) are indicated. Identified proteins are labeled with arrows.

The secretome of EPEC E2348/69 is dominated by the presence of the translocators, EspA, EspB and EspD [9,21]. The genes encoding these proteins are located together in the *LEE4 *operon. To identify less abundant proteins in the EPEC E2348/69 supernatant, we generated an Δ*espADB *mutant and compared the secreted protein profile of this mutant with that of a Δ*escF *T3SS mutant EPEC ICC171 by two dimensional gel electrophoresis (2-DGE). *escF *encodes the needle structure of the LEE-encoded T3SS and mutations in *escF *abolish secretion of the translocator and effector proteins [14,22]. An *escF *mutant was used in preference to *escN*, which encodes the T3SS ATPase, as an *escN *mutant showed greater cell lysis in culture during growth in hDMEM (data not shown). However some cell breakdown was still observed for ICC171 which may account for some spots visualized by 2-DGE (Fig. 1). Both the Δ*espADB *mutant and ICC171 were grown in HEPES buffered DMEM (hDMEM) pH 7.4–7.7 to an OD_600 _of 1.0 to induce expression of the LEE T3SS. Cultures (20 × 5 ml) were pooled to control for variations in growth and supernatant proteins were collected by trichloroacetic acid (TCA) precipitation. Following 2-DGE, consistent and dominant spots were excised for tryptic in-gel digestion and MALDI-TOF mass spectrometry analysis. Since the genome sequence of EPEC E2348/69 was not yet annotated, peptide mass fingerprints were analyzed against a limited library of open reading frames from EPEC E2348/69 http://www.sanger.ac.uk/Projects/Microbes. As expected, the autotransporter EspC was present in the supernatant of both the Δ*espADB *mutant and ICC171. Although we did not identify any non-LEE encoded effector proteins using this approach, we did find that FliC was present abundantly in the supernatants of the Δ*espADB *mutant (Fig. 1) and wild-type EPEC (data not shown) but was greatly reduced in the supernatant of the Δ*escF *mutant, ICC171 (Fig. 1). This was unexpected as previous studies have reported that EPEC flagellation and motility is down regulated by growth in DMEM [23,24]. In addition, we observed that FimA export was upregulated in the Δ*espADB *mutant. Although we did not investigate the basis for increased FimA protein, *fimA *is know to be co-regulated with flagella biosynthesis in *E. coli *[25]. Thus increased FimA production and export may be connected to increased FliC production and export. Unfortunately, we were not able to identify any further protein spots by MALDI-TOF analysis other than FimA, EspC and FliC.

### The LEE-encoded T3SS promotes flagellin export

**Figure 2:**
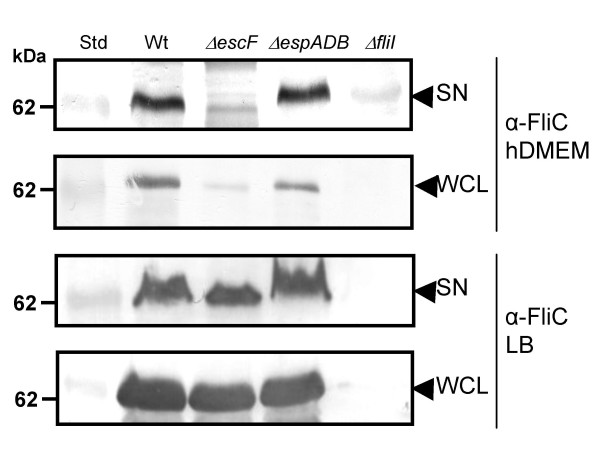
**Immunoblot analysis of secreted proteins in the culture supernatant (SN) and whole cell lysates (WCL) prepared from derivatives of EPEC E2348/69 grown in hDMEM and LB**. Arrows indicate a reactive band corresponding to FliC detected with anti-H6 FliC antibodies.

The reduced amount of FliC in the supernatant of ICC171 grown in hDMEM but not the Δ*espADB *mutant suggested that either flagellin synthesis and/or export was connected to expression of the LEE-encoded T3SS, since both mutants contain a functional flagella biosynthesis locus, or perhaps that the inactivation of *espD *led to increased FliC expression which has been reported previously [23,24]. To examine the association between the presence of a functional LEE-encoded T3SS and flagellin synthesis and export in hDMEM, we used mono-specific anti-H6 FliC antibodies and immunoblotting to examine the production and secretion of FliC into the culture supernatant by various derivatives of EPEC. Initially we confirmed that FliC secretion in hDMEM by ICC171 was reduced compared with EPEC E2348/69 and the Δ*espADB *mutant (Fig. 2). To determine if secretion could occur in the absence of a functional flagella biosynthesis apparatus, we inactivated *fliI *which encodes the flagella system ATPase essential for FliC export by this pathway [26]. Although the results showed that FliC was not found in the supernatant of the Δ*fliI *mutant grown in hDMEM (Fig. 2), a band corresponding to FliC was also not present in whole cell lysates preparations suggesting that mutation of *fliI *also abrogated expression of FliC (Fig. 2). In addition, we observed a reduction in the production of FliC by the *escF *mutant grown in hDMEM (Fig. 2). These data are consistent with previous reports that *fliC *expression is down regulated if the protein is not exported [27]. When EPEC derivatives were grown in LB which promotes motility and down regulation of the LEE-encoded T3SS, FliC was produced and exported by all strains except the *fliI *mutant (Fig. 2). This indicated that mutation of *escF *did not affect *fliC *expression and FliC export under conditions that promote flagellation and motility but suggested that under conditions favoring expression of the LEE-encoded T3SS, *escF *was needed for FliC synthesis and/or export.

### Secretion of flagellin via the LEE-encoded T3SS of EPEC E2348/69

**Figure 3:**
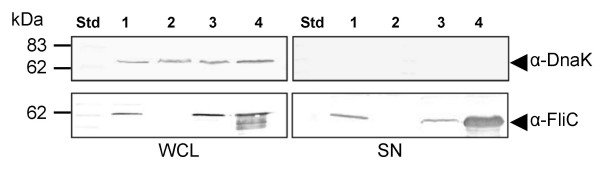
**Immunoblot analysis of secreted proteins (SN) and whole cell lysates (WCL) prepared from derivatives of EPEC E2348/69 grown in hDMEM**. Lane 1: E2348/69; lane 2: Δ*fliC*; lane 3: Δ*fliC *(pFliC) non-induced; lane 4: Δ*fliI *(pFliC) induced with 1 mM IPTG for 30 min. Arrows indicate position of a reactive band corresponding to FliC detected with anti-H6 FliC antibodies or DnaK detected with anti-DnaK antibodies.

To define further the relationship between FliC secretion in hDMEM and expression of the LEE T3SS, we expressed *fliC *from an IPTG inducible promoter in the expression vector, pTrc99A to overcome the negative feedback inhibition of FliC production in the *fliI *and *escF *mutants observed earlier. This plasmid was termed pFliC. A Δ*fliC *mutant was constructed to serve as a control strain and inducible expression and successful secretion of FliC was demonstrated from pFliC 30 min after induction with IPTG (Fig. 3). An analysis of culture supernatants for the presence of the cytoplasmic protein, DnaK, showed that overexpression of FliC from pFliC did not result in increased cell lysis (Fig. 3).

**Figure 4:**
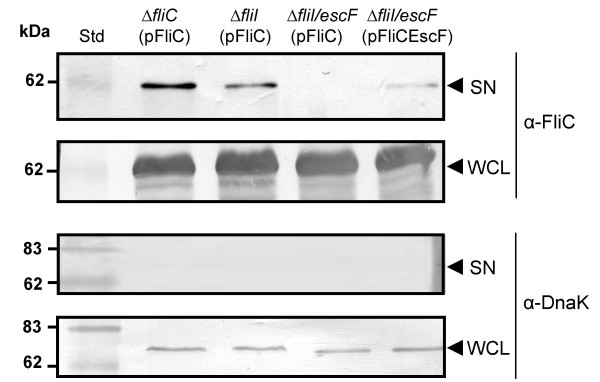
**Immunoblot analysis of secreted proteins (SN) and whole cell lysates (WCL) prepared from derivatives of EPEC E2348/69 grown in hDMEM**. Arrows indicate position of a reactive band corresponding to FliC detected with anti-H6 FliC antibodies or DnaK detected with anti-DnaK antibodies. FliC expression was induced in vitro with 1 mM IPTG from the *trc *promoter in pTrc99A.

To investigate the contribution of the LEE-encoded T3SS and the flagella secretion system to FliC export in hDMEM, we constructed a Δ*fliI*/*escF *double mutant where both the LEE-encoded and flagella secretion systems were inactivated. pFliC was introduced into the Δ*fliC*, Δ*fliI *and Δ*fliI*/*escF *mutant strains and immunoblotting of whole cell lysates showed that FliC expression was successfully induced (Fig. 4). We then examined the supernatants of the Δ*fliI *and Δ*fliI/escF *mutants carrying pFliC for secretion of FliC after induction with IPTG for 30 min. Secretion of FliC was detected in supernatants derived from the Δ*fliI *mutant but was greatly reduced in the Δ*fliI/escF *mutant (Fig. 4). To verify that a functional LEE T3SS was required for FliC secretion when the flagella export system was inactivated, we complemented the Δ*fliI/escF *mutant with pFliCEscF. Immunoblot analysis of supernatant proteins showed that flagellin export was partially restored to the Δ*fliI/escF *mutant upon trans-complementation with *escF *(Fig. 4). The success of trans-complementation with *escF *was also confirmed by performing a fluorescence actin staining (FAS) test (data not shown). Therefore, in the absence of a functional flagella secretion apparatus (due to inactivation of *fliI*), FliC export still occurred if the LEE-encoded T3SS was intact. The involvement of the flagellin chaperone, FliS, in FliC secretion by the LEE-encoded T3SS was examined by constructing a double Δ*fliI/fliS *mutant. Flagellin expressed from pFliC was secreted by theΔ*fliI/fliS *mutant in equivalent amounts to Δ*fliI *(pFliC) suggesting that the FliS chaperone was not involved in LEE-dependent FliC secretion (data not shown). To determine whether FliC was recognized as an effector or a translocator by the LEE-encoded T3SS, we also examined FliC export by a *sepL *mutant. The mutation of *sepL *leads to preferential secretion of effectors and reduced secretion of translocators [28,29]. We found that the *sepL *mutant secreted flagellin in equivalent amounts to the Δ*espADB *mutant suggesting that FliC was recognized as an effector of the LEE-encoded T3SS (data not shown).

### Flagellin exported by the LEE T3SS induces NF-kappa B activity but does not confer motility

**Figure 5:**
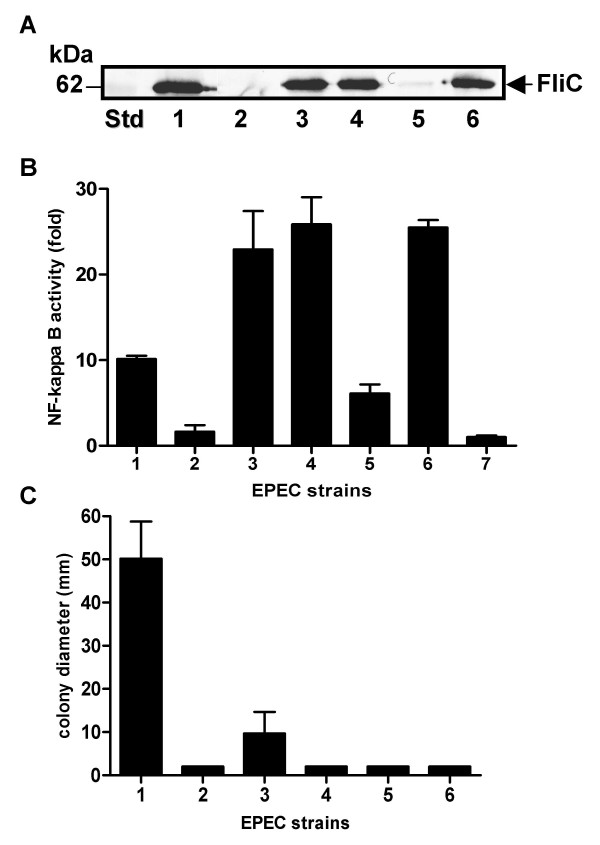
**A. Representative immunoblot of secreted proteins prepared from derivatives of EPEC E2348/69 grown in hDMEM and detected with anti-H6 FliC antibodies**. Lane 1: E2348/69; lane 2: Δ*fliC*; lane 3: Δ*fliC *(pFliC); lane 4: Δ*fliI *(pFliC); lane 5: Δ*fliI*/*escF *(pFliC); lane 6: Δ*fliI*/escF (pFliCEscF). **B**. NF-kappa B-dependent luciferase reporter activity in HEK293 cells stimulated with secreted proteins prepared from derivatives of EPEC E2348/69. 1. EPEC E2348/69; 2. Δ*fliC*; 3. Δ*fliC *(pFliC); 4. Δ*fliI *(pFliC); 5. Δ*fliI*/*escF *(pFliC); 6. Δ*fliI*/*escF *(pFliCEscF); 7. hDMEM alone. Results are expressed as the mean fold increase ± SEM with respect to the unstimulated control (fold = 1) and are representative of three independent experiments performed in triplicate **C**. Motility of derivatives of EPEC E2348/69 shown in (A) in 0.2% hDMEM agar. 1. EPEC E2348/69; 2. Δ*fliC*; 3. Δ*fliC *(pFliC); 4. Δ*fliI *(pFliC); 5. Δ*fliI*/*escF *(pFliC); 6. Δ*fliI*/*escF *(pFliCEscF).

Previous work has shown that FliC from EPEC E2348/69 can stimulate proinflammatory cytokine production through TLR5 signaling [30]. Indeed, EPEC H6 flagellin is a potent activator of interleukin-8 release in T84 and HT-29 intestinal epithelial cells [24,31]. Here we investigated host cell signaling in response to EPEC E2348/69 flagellin by measuring NF-kappa B activation in human embryonic kidney HEK293 cells using an NF-kappa B dependent luciferase reporter assay. Since HEK293 cells possess functional TLR5 and non-functional forms of TLR2 and TLR4, the cell line is most likely responsive only to flagellin and not to Gram-negative lipoproteins and lipopolysaccharide [32]. As expected, there was a correlation between the presence of FliC in the bacterial culture supernatant and NF-kappa B activation (Fig. 5). Although the activation of NF-kappa B by wild type EPEC E2348/69 supernatant proteins (Fig. 5B) appeared lower than strains producing the same amount of FliC (Fig. 5A), the western blot presented represented one experiment only and NF-kappa B activation was performed more than three times using different preparations of supernatant proteins. Despite batch to batch variation in supernatant protein preparations, the results of mean NF-kappa B activity showed that FliC present in the culture supernatant of the Δ*fliI *mutant carrying pFliC could stimulate a 5 fold greater increase in NF-kappa B activity compared to the Δ*fliI/escF *mutant carrying pFliC (*P *= 0.0042, unpaired two tailed *t*-test). As expected, the *fliI *mutant derivatives of EPEC E2348/69 secreting FliC via the LEE-encoded T3SS were non-motile (Fig. 5C), due to the absence of an intact flagella export apparatus.

## Discussion

Many Gram-negative pathogens utilize a T3SS to deliver diverse effector proteins directly into eukaryotic cells. The structure of the T3SS apparatus is conserved among different pathogens and shares structural similarity with the flagella basal body. The reported ancestral relationship between the two secretion systems is based on low sequence similarity between some components as well as functional conservation [33]. Under certain conditions, virulence effector proteins may be secreted, but not translocated by the flagella T3SS [34-37]. The preferential secretion of effector proteins by their cognate T3SS rather than the flagella export apparatus depends largely on a system of chaperones that confer pathway specificity. In *Salmonella enterica *serovar Typhimurium, truncated forms of the effectors SptP and SopE that lack the chaperone binding domain for secretion by the T3SS are instead secreted by the flagella export apparatus [35,38]. This suggests that not only do the T3SS system chaperones confer pathway specificity, but also that the flagella export system is the default secretion pathway for unchaperoned proteins [35].

Recently, Miao *et al *(2006) showed that flagellin from *S. *Typhimurium present in the cytosol of infected macrophages stimulated IL1-β release in macrophages through activation of the intracellular NACHT-leucine-rich repeat protein, Ipaf. The activation of Ipaf by cytosolic flagellin was dependent on the SPI1-encoded T3SS and not the flagella biosynthesis locus [39]. The authors suggested that flagellin secretion most likely occurred via the SPI1 T3SS needle complex and concluded that the accidental export of flagellin into the host cell by the SPI1 T3SS may provide an advantage to the host through the stimulation of pro-inflammatory cytokine expression and release [39]. In this study, we also detected the secretion of flagellin by EPEC in the absence of a functional flagella export apparatus that was largely dependent on the LEE-encoded T3SS and this indiscriminate secretion of flagellin had the potential to stimulate NF-kappa B activity. However, we were not able to visualize FliC in the intracellular environment of the host cell using immunofluorescence to compare FliC staining in permeabilized and non-permeabilized HeLa cells infected with EPEC (data not shown). This suggested that in contrast to the SPI1-encoded T3SS of *Salmonella*, the LEE-encoded T3SS of EPEC did not translocate flagellin into the host cell. It remains possible however, that the method used here to visualize intracellular flagellin was not sensitive enough to detect small amounts of translocated FliC protein.

## Conclusion

We conclude that the flagella and LEE-encoded T3SSs of EPEC have undergone selection to evolve temporal differences in expression and specificity of function through a system of chaperones and regulatory checks that maintain mutually exclusive export of the T3SS effectors and flagellin. The fact that EPEC infection does not result in a strong inflammatory response suggests that there has been strong evolutionary selection against TLR5 activation during A/E lesion formation [40]. Indeed, despite the structural similarity between EspA and FliC, EspA lacks the major D0 domain that activates TLR5 signaling by FliC [41]. The dedicated function of the respective virulence-associated and flagella T3SSs to the secretion of their cognate substrates is likely to be critical in ensuring that flagellin is not accidentally released during the important initial stages of infection where it may prematurely activate inflammatory signaling pathways.

## Methods

### Bacterial strains, cell lines and growth conditions

The bacterial strains used in this study are listed in Table 1. *E. coli *strains were grown overnight at 37°C in Luria Bertani (LB) broth followed by culturing in 25 mM HEPES-buffered DMEM with 44 mM NaHCO_3 _(hDMEM). HeLa cells and HEK293 cells were cultured at 37°C in the presence of 5% CO_2 _in DMEM supplemented with 10% FCS and 2 mM glutamine. Where necessary the following antibiotics were supplied at the following final concentrations: kanamycin (100 μg/ml), chloramphenicol (25 μg/ml) and ampicillin (100 μg/ml).

**Table 1:** Bacterial strains and plasmids used in this study

Strain/plasmid	**Characteristic(s)**^a^	Motility In DMEM	**FAS**^b^	Reference
Strains				
E2348/69	Wild type EPEC O127:H6	+	+	[47]
ICC171	E2348/69 Δ*escF *(Kan^R^)	+/-	-	[14]
EPEC Δ*espADB*	E2348/69 Δ*espADB *(Kan^R^)	+	-	This study
EPEC Δ*fliI*	E2348/69 Δ*fli *(Cm^R^)	-	+	This study
EPEC Δ*fliC*	E2348/69 Δ*fliC *(Cm^R^)	-	+	Grubman *et al*, submitted
EPEC Δ*fliI*/*escF*	E2348/69 Δ*fliI/escF *(Cm^R^, Kan^R^)	-	-	This study
EPEC Δ*fliI/fliS*	E2348/69 Δ*fliI/fliS *(Cm^R^, Kan^R^)			This study
EPEC Δ*sepL*	E2348/69 Δ*sepL *(Kan^R^)			[48]
Plasmids				
pKD46	Red Recombinase helper plasmid (Amp^R^)	N.A^c^	N.A	[45]
pKD3	Red Recombinase template plasmid (Cm^R^)	N.A	N.A	[45]
pKD4	Red Recombinase template plasmid (Kan^R^)	N.A	N.A	[45]
pTrc99A	High-copy number expression vector (Amp^R^)	N.A	N.A	[49]
pFliC	Derivative of pTrc99A encoding *fliC *from EPEC E2348/69 (Amp^R^)	N.A	N.A	This study
pFliCEscF	Derivative of pTrc99A encoding *fliC *and *escF *from EPEC E2348/69 (Amp^R^)	N.A	N.A	This study
pCDNA3	Eukaryotic expression vector	N.A	N.A	Promega

^a^Kan, kanamycin; Cm, chloramphenicol; Amp, ampicillin.

^b^FAS, Fluorescent actin staining test.

^c^N.A., not applicable.

### Isolation of secreted proteins

EPEC was inoculated into 5 ml of LB and grown overnight at 37°C with shaking. EPEC was routinely diluted 1:100 in DMEM containing 44 mM NaHCO_3 _buffered with 25 mM HEPES and grown at 37°C with shaking. Bacterial supernatants were analyzed at mid- to late-log phases of growth [42]. To ensure removal of bacteria and cellular debris, the bacterial supernatants were filtered through 0.45 μm pore filters (Millipore, Bedford, MA) [43]. The cell-free supernatants were precipitated with a final 10% w/v trichloroacetic acid (TCA) solution and subsequent centrifugation at 13,000 rpm for 45 min at 4°C followed by three methanol washes. Equal amounts of proteins were analyzed by SDS-PAGE and by two-dimensional gel electrophoresis. Proteins of interest were subjected to mass spectrometry.

### SDS-PAGE and immunoblotting

The bacterial suspensions were adjusted to an absorbance of 1.0 at OD_600_. Equal numbers of bacteria were used to prepare whole cell extracts in sample denaturation buffer and separated by 12% SDS-PAGE. The gels were stained with Coomassie Brilliant Blue R-250 (Bio-Rad, Hercules, CA) or transferred onto nitrocellulose membranes (Pall Life Science, Pensacola, FL) for immunoblotting. The immobilized proteins were incubated with primary antibodies against H6 flagellin (Statens Serum Institut, Denmark) or cytoplasmic protein DnaK (Assay Designs, Ann Arbor, MI) followed by incubation with goat anti-rabbit (Sigma, St. Louis, MO) or sheep anti-mouse IgG (Chemicon, Melbourne, Australia) conjugated to alkaline-phosphatase. Antibody binding was detected with chemiluminescent reagent (Astral Scientific, Gymea, NSW, Australia).

### Two-dimensional Gel Electrophoresis

Proteins secreted from approximately 10^9 ^cells (~120 μg) were precipitated with a final 10% w/v TCA solution and material was resuspended in 460 μl of following sample solution: 5 M urea (Amersham Pharmacia Biotech, Sweden), 2 mM tributylphosphine (TBP) 2% CHAPS, 2% (v/v) carrier ampholytes (Bio-Rad, CA, USA), 2% SB 3–10 or 2% SB 4–7 and trace of bromophenol blue (Pharmacia Biotech) by vortexing [44]. Insoluble material was removed by centrifugation at 12 000 × *g *for 10 min. The 460 μl samples were used to passively rehydrate pH 3–10 or pH 4–7 immobilized pH gradient dry strips for 18 h at room temperature (Bio-Rad). Isoelectric focusing was performed using a step-wise protocol with a final voltage of 3500 V on a Multiphor II (Amersham Pharmacia Biotech, Uppsala, Sweden) equaling a total of 75 kV h. The second dimension was performed on 12% SDS-PAGE gels using a Protean II Multi-Cell (Amersham Pharmacia). The gels were stained with Colloidal Coomassie Blue G-250 or Sypro Ruby (Molecular Probes, Eugene, OR). Protein samples were isolated from at least three independent preparations of 20 × 5 ml cultures. More than three separate gels were analyzed for each sample. Protein spots that displayed dominant and consistent patterns were selected for further identification.

### Matrix-assisted laser desorbtion/ionization time of flight (MALDI-TOF) mass spectrometry

Protein spots were excised from gels and washed with 50 mM ammonium bicarbonate/100% acetonitrile (60:40 v/v). The gel pieces were dried and rehydrated in a solution containing sequencing grade modified trypsin (Promega, Madison, WI) for 1 h at 4°C. Excess trypsin solution was removed and the rehydrated gel pieces were immersed in 50 mM ammonium bicarbonate and incubated overnight at 37°C. Eluted peptides were concentrated and desalted using μ-C_18 _Zip-Tips™ (Millipore Corp., Bedford, MA) and trifluoroacetic acid in acetonitrile solutions. Mass spectra were acquired at the Monash University proteomics facility by Dr. Simon Harris. Lists of mono-isotopic peaks corresponding to various peptides were generated manually. Peptide masses were searched against the NCBInr database by use of the MASCOT software (Matrix Science), with the mass tolerance set to 50 ppm or 200 ppm. Proteins with sequence coverage exceeding 20% with the matched proteins were considered positive for identification.

### Construction of non-polar mutants of EPEC E2348/69

Non-polar mutations of *espADB, fliC*, *fliI *were constructed in EPEC E2348/69 using the λ Red recombination system [45]. In addition, double mutants of *fliIfliS *and *fliIescF *were created using alternative antibiotic selection markers. Mutations were obtained using pKD3 as a template with the primer pairs: *fliC*_Δ_F/*fliC*_Δ_R and *fliI*_Δ_F/*fliI*_Δ_R and pKD4 as a template with *fliS*_Δ_F/*fliS*_Δ_R and *espADB*_Δ_F/*espADB*_Δ_R (Table 2). The PCR products were digested with *Dpn*I before being electroporated into EPEC E2348/69 carrying the Red Recombinase expression plasmid, pKD46. Mutants were selected on LB plates supplemented with chloramphenicol or kanamycin. All mutations were confirmed by PCR using primers flanking the targeted region (designated "verify", Table 2) and primers within the chloramphenicol or kanamycin resistance gene.

**Table 2:** Oligonucleotide primers used in this study

Primer	Sequence (5'-3')
*fliC_Δ_*F	CGTAATCAACGACTTGCAATATAGGATAACGAATCATGGCACAAGTCAT TAATACCTGTGTAGGCTGGAGCTGCTTCG
*fliC_Δ_*R	GCATCAGGCAATTTGGCGTTGCCGTCAGTCTCAGTTAATCAGGTTACAAC GACATATGAATATCCTCCTTA
*fliC*_verify_F	GAGTATTTCGGCGACTAAC
*fliC*_verify_R	GCAGGGTTTTAATCGGACG
*fliI_Δ_*F	CCAGGAGTGGTGTAATGACCACGCGCCTGACTCGCTGGCTAACCACGCTG GTGTGTAGGCTGGAGCTGCTTCG
*fliI_Δ_*R	GGAGAGACGCTTCCCAGTCCGCGCGTTCAAAAATGCCTTGTTGCAAATAG CCCATATGAATATCCTCCTTA
*fliI*_verify_F	GCTGGCAAGAACTCTGCCG
*fliI*_verify_R	GACACTGTCGGGAAAATACGC
*fliS_Δ_*F	GCAGTTCGAAAACAACAGTAATTCCAAGTAATCATTATTCATCGGGAGAC AGGTCTGTGTAGGCTGGAGCTGCTTCG
*fliS_Δ_*R	GCTGGCTTTTTTCGACGAGTTGTTGCCAGGCGAAATATAAATGCGGTGCAT GGTTCACATATGAATATCCTCCTTA
*fliS*_verify_F	CACGACCACCATCGGCAG
*fliS*_verify_R	GAATCAGGCGCAGCATCGGGC
*fliC*F	CATGCCATGGCACAAGTCATTAATACC
*fliC*R	CGCGGATCCTTAACCCTGCAGCAGAGAC
*escF*F	GCTCTAGATCTGAGGGAAATTTAATG
*escF*R	CCCAAGCTTTTAAAAACTACGGTTAGA
*espADB_Δ_*F	GTCATGCTAAGAAAGATTATGAAGAGGTATATACATGGATACATCAACT ACAGCTGTGTAGGCTGGAGCTGCTTCG
*espADB_Δ_*R	GTTCAAGATAGTAATTAAACACTTCATCATTAAACGTATCGACCATGATC AACATATGAATATCCTCCTTA
*espADB*_verify_F	CCTTCTCGGGTATCGATTGTCG
*espADB*_verify_R	CGTTAAGCCCTGTTTGGTTACG
pTrc99AF	CGGTTCTGGCAAATATTC

### Construction of FliC and EscF expression vectors

A 1643 bp fragment and a 237 bp fragment containing the *fliC *and *escF *genes, respectively, were amplified by PCR using primers *fliC*F and *fliC*R (*fliC*) and *escF*F and *escF*R (*escF*). Genomic DNA from EPEC E2348/69 was used as a template in PCR (Table 2). The PCR products were digested with *Nco*I/*Bam*HI and *Xba*I/*Hind*III for *fliC *and *escF*, respectively, and ligated into pTrc99A, generating expression vectors pFliC and pFliCEscF. Secretion of flagellin was checked by immunoblotting using anti-H6 antibodies and the function of EscF was confirmed by a fluorescent actin staining (FAS) test on HeLa cells infected with an *escF *mutant carrying pFliCEscF.

### Fluorescence actin staining test

FAS test was performed on infected HeLa cells as described previously [46]. Briefly, EPEC strains were grown overnight at 37°C with shaking in LB. Bacteria were added to cells at a dilution factor of 1:50 in DMEM and incubated at 37°C for 3–5 hours. Cells were fixed in 1% paraformaldehyde, permeabilized with 0.1% Triton-X 100 (Sigma) and stained with 0.5 μg/ml Phalloidin-TRITC (Sigma). Coverslips were mounted in DAKO fluorescent mounting medium (DAKO Corporation Carpinteria, USA) and stored at 4°C in the dark. Samples were examined under a 100× objective using an Olympus, BX51 epifluorescence microscope. Images were acquired using an Olympus DP-70 digital camera and merged using DP controller software version 1.1.1.71.

### Cell transfection and NF-kappa B luciferase assays

NF-kappa B luciferase reporter activity in response to the proteins secreted by EPEC strains was determined in HEK293 cells, as described elsewhere [32]. In brief, cells were seeded in 96 well plates (Iwaki, Tokyo, Japan) at 2 × 10^4 ^cells per well. Each well was transfected with 230 ng of DNA consisting of 60 ng Igkappa-luciferase reporter construct and pCDNA3 complexed with Fugene (Roche, Basel, Switzerand) and made to 100 μl with cell culture medium. One day post transfection, the cell culture media was replaced and cells were stimulated for 4 h with 10 μg of TCA precipitated proteins in PBS. At the completion of the HEK293 co-culture experiments, culture media was removed and cells were lysed using reporter lysis buffer (Promega, Madison, WI). Luminescence was measured using a FLUOstar Optima luminometer (BMG Labtech, Offenburg, Germany). All samples were measured in triplicate and all experiments were performed at least three times.

### Motility assay

Freshly grown bacterial colonies were stabbed into motility agar (0.2% agar) plates. The plates were incubated face up at 37°C for 16 to 24 h, and motility was assessed by measuring the migration of bacteria through the agar by zone of growth. Results are expressed (in mm) as the mean ± standard deviation of triplicate colonies from 3 independent experiments.

## Abbreviations

EPEC: enteropathogenic *E. coli*; LEE: locus of enterocyte effacement; T3SS: type III secretion system; A/E: attaching and effacing lesion; TLR: toll-like receptor; SEM standard error of the mean.

## Authors' contributions

LB participated in the design of the study, carried out the experiments and drafted the manuscript. SAB participated in the interpretation of results and writing of the manuscript. MK supervised experiments and participated in writing the manuscript. RLF and ELH participated in the design of the study, the interpretation of results, the writing of the manuscript, the supervision of LB and MK and supplied funding for the project. All authors have read and approved the final manuscript.
